# The feasibility of a multidimensional intervention in lymphoma survivors with chronic fatigue

**DOI:** 10.1007/s00520-023-08204-5

**Published:** 2023-12-14

**Authors:** SKH Bøhn, LM Oldervoll, KV Reinertsen, M Seland, A Fosså, C Kiserud, T Skaali, TS Nilsen, R Blomhoff, HB Henriksen, HC Lie, T Berge, E Fjerstad, T Wisløff, M Slott, I Zajmovic, L Thorsen

**Affiliations:** 1 National Advisory Unit for Late Effects After Cancer Treatment, Department of Oncology, Oslo University Hospital, Radiumhospitalet, Oslo, Norway; 2 Centre for Crisis Psychology, Faculty of Psychology, University of Bergen, Bergen, Norway; 3 Department of Public Health and Nursing, Faculty of Medicine and Health Sciences, Norwegian University of Science and Technology, Trondheim, Norway; 4 Department of Clinical Service, The Cancer Rehabilitation Center, Aker, Oslo University Hospital, Oslo, Norway; 5 Institute of Physical Performance, Norwegian School of Sports Sciences, Oslo, Norway; 6 Department of Nutrition, Institute of Basic Medical Sciences, University of Oslo, Oslo, Norway; 7 Department of Clinical Service, Division of Cancer Medicine, Oslo University Hospital, Oslo, Norway; 8 Department of Behavioural Medicine, Institute of Basic Medical Sciences, Faculty of Medicine, University of Oslo, Oslo, Norway; 9 Diakonhjemmet Hospital, Oslo, Norway; 10 Health Services Research Unit, Akershus University Hospital, Lørenskog, Norway

**Keywords:** Lymphoma survivors, Chronic fatigue, Exercise, Nutrition, Cognitive behavioural therapy, Patient education

## Abstract

**Purpose:**

Chronic fatigue (CF) affects 25–30% of lymphoma survivors, but interventions designed to reduce fatigue are lacking. The main aim of this study was to test the feasibility of a multidimensional intervention study in lymphoma survivors with CF. Secondary aims were to describe individual changes in fatigue, quality of life (QoL) and physical performance from pre (T0) to post (T1) intervention.

**Methods:**

This feasibility study was as a one-armed intervention study performed in 2021. Hodgkin or aggressive non-Hodgkin lymphoma survivors received mailed study information and Chalder Fatigue Questionnaire and were asked to respond if they suffered from fatigue. The 12-week intervention included patient education, physical exercise, a cognitive behavioural therapy (CBT)-based group program and nutritional counselling. Feasibility data included patient recruitment, completion of assessments, adherence to the intervention and patient-reported experience measures. Participants responded to questionnaires and underwent physical tests at T0 and T1.

**Results:**

Seven lymphoma survivors with CF were included. Of all assessments, 91% and 83% were completed at T0 and T1, respectively. Adherence to the interventional components varied from 69% to 91%. At T1, all participants rated exercise as useful, of whom five rated the CBT-based program and five rated individual nutritional counselling as useful. Five participants reported improved fatigue, QoL and physical performance.

**Conclusion:**

Lymphoma survivors with CF participating in a multidimensional intervention designed to reduce the level of fatigue showed high assessment completion rate and intervention adherence rate. Most of the participants evaluated the program as useful and improved their level of fatigue, QoL and physical performance after the intervention.

**Trial registration:**

ClinicalTrials.gov*, identifier:* NCT04931407. Registered 16. April 2021-Retrospectively registered. https://www.clinicaltrials.gov/ct2/show/NCT04931407

**Supplementary Information:**

The online version contains supplementary material available at 10.1007/s00520-023-08204-5.

## Background

Malignant lymphomas are among the most common malignancies affecting young individuals, with a median age at diagnosis of 45 and 70 years for Hodgkin- and non-Hodgkin lymphoma, respectively [1]. The 5-year relative survival rate has reached 90 % for Hodgkin lymphoma and about 80 % for non-Hodgkin lymphoma. Since lymphoma patients are relatively young at diagnosis and the cure rates are overall high, a large proportion live for many years, up to decades, after treatment [1]. Thus, lymphoma survivors are susceptible to several treatment-induced late effects, including chronic fatigue (CF), neuropathy, mental distress, second cancer and cardiovascular morbidity [2–6].

Fatigue, defined as a “distressing, persistent, subjective sense of physical, emotional, and/or cognitive tiredness or exhaustion related to cancer or cancer treatment that is not proportional to recent activity and interferes with daily functioning”, is one of the most prevalent and distressing symptoms during cancer treatment [7]. For most patients, the level of fatigue decreases during the first months after treatment, but for a significant proportion, the level of fatigue remains high for years [8]. Substantial fatigue lasting for 6 months or longer has been defined as CF [9, 10]. Studies using this definition have shown that the prevalence of CF is 25–30% among lymphoma survivors, with a large negative impact on their work ability and quality of life, causing severe individual and/or societal consequences [2, 3, 11].

Randomized controlled trials (RCTs) have shown that physical exercise and psychological interventions alone or in combination reduce the level of fatigue during and shortly after cancer treatment in several diagnostic groups [7, 12–16]. Moreover, current evidence suggests associations between nutritional status and diet and the level of fatigue among cancer survivors [17]. However, the majority of these studies are conducted in breast cancer patients or populations with mixed cancer diagnoses who are undergoing or have recently completed treatment, and none of the studies have CF as an inclusion criterion for study entry [14]. RCTs investigating the effect of multidimensional interventions including patient education, physical exercise, psychological interventions and nutritional counselling among long-term cancer survivors with CF are lacking [14]. Considering the multifactorial nature of CF, we believe that a multidimensional intervention is more effective than single-focused interventions. However, it is unknown whether a comprehensive intervention is of interest and/or is feasible among cancer survivors with CF, especially considering that these survivors already have a low energy level.

Therefore, before launching a full-scale RCT to evaluate the effects of a multidimensional intervention, including both patient education, physical exercise, a cognitive behavioural therapy (CBT)-based program and individual nutritional counselling among lymphoma survivors with CF, feasibility issues need to be properly addressed. In addition, it is important to explore whether selected outcome measures capture changes from pre to post intervention in order to estimate a correct sample size in a future RCT.

The main aims of the present feasibility study are therefore to assess patient recruitment and retention, assessment completion rate and adherence rate to a multidimensional intervention in lymphoma survivors with chronic fatigue (CF). Secondary aims are to describe individual changes in fatigue, health-related quality of life (HRQoL), physical performance and dietary intake from pre to post intervention.

## Methods/design

This feasibility study was a one-armed intervention study performed from March to June 2021 (Fig. 2). Hodgkin or aggressive non-Hodgkin lymphoma survivors, aged > 18–65 years at diagnosis and who terminated curatively intended treatment in January to May 2014 or July to December 2019 (2 or 7 years before inclusion), were identified through the Lymphoma registry at Oslo University Hospital. Ages > 18–65 years were chosen because we wanted to focus on an adult population of working age. Survivors with indolent lymphoma, ongoing cancer treatment, relapse or second cancer were not eligible.

### Inclusion procedure

All identified survivors, regardless of whether they had CF or not, received written study information and Chalder Fatigue Questionnaire (FQ) by mail. Those who perceived themselves as suffering from fatigue, and who were interested in participating, were asked to complete and return FQ [9]. Those with CF, measured by FQ, were contacted by phone for additional study information. Those still interested and able to participate were scheduled for a medical screening for final participation approval.

### Medical screening

The medical screening was performed by video or telephone consultation with an oncologist. The screening included a review of the participant’s medical- and fatigue history and evaluation of blood test results (including haematology, tests of liver, kidney, thyroid and gonadal function, and vitamins investigating nutritional status). Survivors who reported fatigue symptoms as present more than 1 year before cancer diagnosis, with abnormal blood tests indicating a somatic condition requiring immediate treatment likely to reduce the level of fatigue or with known somatic and/or mental disorders (e.g. severe heart failure/disease, lung disease, use of wheelchair/crutches, dementia, severe depression, schizophrenia, substance abuse disorder), were excluded.

### The multidimensional intervention

**Fig. 1:**
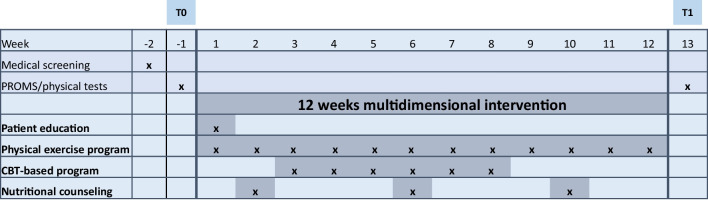
Design of the multidimensional intervention. T0, pre intervention; T1, post intervention. PROMs, patient-reported outcome measures*: the Fatigue Questionnaire and European Organization for Research and Treatment of Cancer Quality of Life Questionnaire. CBT, cognitive behavioural therapy

The intervention lasted for 12 weeks and included four components: patient education, physical exercise, a CBT-based group program and individual nutritional counselling (Fig. 1). The patient education, CBT-based group sessions and individual nutrition counselling were performed as digital sessions led by the staff at the Cancer Rehabilitation Centre at Oslo University Hospital, while the physical exercise was supervised in-person by a physiotherapist in each participant’s local municipality. Each component of the intervention was developed based on published studies on single-targeted interventions [14] and the clinical experiences of the staff at the Cancer Rehabilitation Centre, who were already offering all components of the multidimensional intervention as separate programs for cancer survivors with fatigue.

The intervention started with a 2-h digital *patient education* session held by health personell at the Cancer Reahabilitation Centre covering the following topics related to CF: CF after lymphoma (by oncologist), exercise and recovery (by physiotherapist), coping strategies (by psychologist) and diet and nutrition (by registered clinical dietitian).

The *physical exercise program* included two weekly sessions for 12 weeks: one supervised by physiotherapist in the local municipality and one unsupervised. The s*upervised one-to-one sessions* lasted 60–75 min: 10–15 min warm-up at low intensity, followed by four 2-min aerobic intervals at moderate intensity, with active rest for 2 min between the intervals, and 10–20 min of resistance training with six exercises (i.e. variants of squats, push-ups, standing rowing, Arnold press, seat-lift and dead bugs). At the end of each supervised session, participants were offered a 20-min digital recorded instruction including body awareness and attentive presence, inspired by Norwegian Psychomotor Physiotherapy (NPMP) [18].

The exercise intensity in both supervised and unsupervised sessions was recorded using heart rate monitors. During warm-up, the intensity was 50–60% of each participant’s peak heart rate (*HR*_peak_) (i.e. the highest heart rate value attained during a pre-intervention maximal treadmill test). During the two first familiarization weeks, the intensity of the aerobic intervals was 60–70% of *HR*_peak_, increasing to 70–75% of *HR*_peak_ in week 3–6 and to 80–85% of *HR*_peak_ from week 7. The resistance training was performed with low or no load the two first weeks to ensure familiarization with the exercises. From weeks 3 to 12, the load was increased to 8–12 repetitions maximum (i.e. the maximum load the participant could lift with good technique). In weeks 1–4, all upper body exercises were performed in one set, and lower body exercises in two sets. From week 5, one additional set was added for all exercises. The participants logged exercise details from the supervised and unsupervised aerobic and resistance exercise sessions in an exercise log. These details included type of activity, duration, number of sets and repetitions of the strength exercise, heart rate during aerobic intervals and perceived exertion (Borg scale). The exercise log was filled out in collaboration with the physiotherapist, to ensure that the exercise data was entered correctly.

In the *unsupervised sessions*, the participants were instructed to copy the content and intensity of the supervised sessions. From week 8, the participants could add a voluntary third weekly session, including a continuous physical activity (i.e. walking, jogging, cross-country skiing, cycling) lasting 30–60 min with moderate intensity (i.e., 70–75% of HR_peak_).

The *CBT-based group program* starting the third week consisted of six digital weekly sessions, led by two clinical psychologists each time. The program was based on a standardized self-management manual primarily developed to target fatigue in patients with inflammatory rheumatic disease [19]. Topics and strategies emphasized in the program were how to: understand CF; make a plan to balance activity and restitution; be a constructive supporter for themselves; and manage unhelpful thoughts, excessive worry and rumination. The program aimed to strengthen perceived self-efficacy in the management of fatigue. The participants used a comprehensive workbook, with assigned homework and practices to implement these mental and behavioural strategies in everyday life. Each group session contained a block were the participants shared their experiences with the homework. However, given their challenges with fatigue and the comprehensive intervention, not completing homework was met with understanding and acceptance from both the psychologists and other participants. Participants were encouraged to meet for each session regardless of completing the homework or not.

The *nutritional counselling* included three individual digital sessions with a registered clinical dietitian in weeks 2, 6 and 10. The counselling was based on each participants dietary intake and nutrition status as reported in the DIGIKOST-FFQ [20, 21]. The aim of the counselling was to improve adherence to the Norwegian Food-Based Dietary Guidelines (NFBDG) [22], which is based on the international guidelines from the World Cancer Research Fund International [23]. The motivational interviewing technique was used to select one to three dietary goals according to the NFBDG.

## Assessments

Assessments of the study included feasibility outcomes, patient-reported outcome measures (PROMs) and physical tests related to individual changes from pre (T0) to post intervention (T1) (Fig. 1).

## Feasibility outcomes

### Recruitment and retention

Recruitment and retention were defined based on the number of responders who perceived themselves as suffering from fatigue among the invited, who were also participants without perceived fatigue. Among the responders, eligible individuals were those with CF defined based on FQ. The number of individuals included among the eligible, and a number of participants who dropped out among the included were assessed. Based on the prevalence of CF among lymphoma survivors [2, 3, 11], we expected that 25–30% of the invited survivors would be eligible for inclusion and that half of these would respond to the invitation.

### Assessment completion rate and intervention adherence rate

The *assessment completion* rate was defined as the sum of all completed assessments divided by the sum of all planned assessments among all participants at T0 and T1. The *intervention adherence rate* was defined as the total number of sessions attended divided by the total number of sessions planned for each component of the intervention among all participants. Adherence to the aerobic exercise was recorded by a modified version of the metrics suggested by Nilsen et al [24], where the prescribed- and actual aerobic training dose was based on interval duration (minutes) multiplied by HR measured by a pulse sensor. For each session, the number of participants completing the aerobic session as planned, more than planned or less than planned is described.

### Adverse events

Any adverse events during exercise were logged by the local physiotherapists and reported to the medical responsible oncologist and principle investigator.

### Patient-reported experience measures (PREMs)

After the intervention, a semi-structured telephone-interview was conducted with each participant. In this interview, the participants were asked to respond to the following pre-defined questions with corresponding response alternatives: (a) How useful did you find each component for improvement of fatigue? (highly useful, useful or less useful), (b) How did the total load of the intervention had influence your fatigue? (it went just fine, it went moderately well or it was too much) and (c) How was the level of your fatigue after the intervention compared to before their participation in the study? (better, no change, worse). Number of responses to each question were then summed up.

## Patient-reported outcome measures (PROMs) and physical tests

### PROMs

The paper questionnaire included the Fatigue Questionnaire (FQ) [9] and European Organization for Research and Treatment of Cancer Quality of Life Questionnaire (EORTC QLQ-C30), version 3 [25, 26]. FQ contains seven items covering physical fatigue and four items covering mental fatigue, during the past month. Each item includes four response alternatives scored from 0 to 3 (0,1,2,3). The physical fatigue score ranges from 0 to 21, the mental fatigue score from 0 to 12, and the total fatigue score from 0 to 33. Higher scores imply more physical/mental/total fatigue. An additional question assesses the duration of fatigue (< 1 week/< 3 months/3–6 months/≥ 6 months). A dichotomized scoring system (0 = 0, 1 = 0, 2 = 1, 3 = 1) of the responses to each item is used for case definition [9]. CF was defined by a dichotomized sum score ≥ 4 and duration for ≥ 6 months.

EORTC QLQ-C30 includes five functioning scales (physical [5 items], role [2 items], emotional [4 items], cognitive [2 items], social [2 items] functioning) and two items measuring global health/QoL. The scores for each scale were transformed to a 0 to 100 scale according to the EORTC scoring algorithms [25]. High functioning and global QoL yield a high score.

Dietary intake and nutrition status in accordance to the NFBDG [22] were assessed by the digital DIGIKOST- food frequency questionnaire (FFQ) [20, 21].

### Physical tests

Physical performance was assessed by physiotherapist in the local municipality and included three tests: indirect maximal treadmill test (modified Balke protocol) assessing cardiorespiratory fitness [27], 30-s sit-to-stand test measuring muscle functioning in lower body and push-ups test assessing muscle strength in upper body [28].

### Statistics

Continuous variables were described by median and range and categorical variables as numbers and percentages. IBM SPSS Statistics version 28.0 (SPSS, Chicago, IL) was used for descriptive statistics. As this was a feasibility study including only seven participants, we did not conduct statistical analyses of the changes in PROMs or physical tests from pre to post intervention.

## Results

### Recruitment and retention

**Fig. 2:**
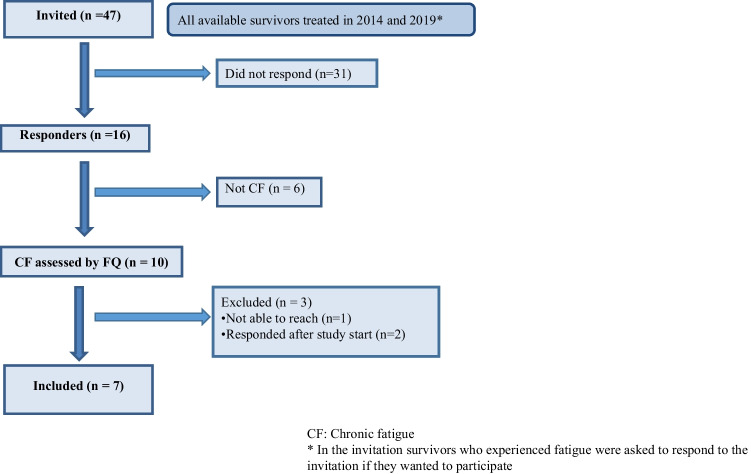
Flow chart

Sixteen of 47 invited survivors responded, i.e. considering themselves as suffering from fatigue. Ten of the 16 responders had CF and were eligible, of whom three were excluded as they were not able to contact (*n* = 1) or responded after study start (*n* = 2). Thus, seven participants were included, six men and one woman (Fig. 2). None dropped out of the study.

The median age of the participants was 53 years (range 31–59), and all were diagnosed with aggressive non-Hodgkin lymphoma, three in 2014 and four during 2019 (Table 1).

**Table 1:** Characteristics of participants (*n* = 7)

Demographic variables	
Current age, median (min. –max.)	53.0 (31–59)
Male/female	6/1
Education > 12 years	4
Living with partner	6
Living with children < 18 years	3
Medical variables	
BMI < 30/≥ 30 kg/m^2^	2/5
Year of treatment completion	
2014/2019	3/4
Lymphoma subtype	
DLCBL*	4
Burkitt lymphoma	1
High-grade B-cell lymphoma	1
Primary CNS lymphoma	1
Ann Arbor Stage	
I–II	3
III–IV	4
Chemotherapy regimen	
R-CHOP	4
GMALL	2
Matrix or R-MPV	1
Any/mediastinal radiation therapy	3/2

*Diffuse large cell B-lymphoma. *R-CHOP* rituximab, cyclophosphamide, doxorubicin hydrochloride (hydroxydaunorubicin), vincristine sulfate (oncovin), and prednisone. *GMALL* German multicenter study group for adult acute lymphoblastic leukemia, *MATRIX* cytarabine-methotrexate-rituximab-thiotepa, *R-MPV* rituximab, methotrexate, procarbazine, and vincristine

### Assessment completion rate

All participants completed the questionnaire at T0 and T1. Six completed the DIGIKOST-FFQ at T0 and five at T1. All participants completed the treadmill test at T0 and five at T1, and six completed the sit-to-stand test and push-up test at T0 and T1. The total completion rates of the questionnaire, DIGIKOST-FFQ and physical tests were 91% at T0 and 83% at T1 (Table 2).

**Table 2:** Number of participants completing each of the assessments at baseline (T0) and immediately after (T1) the intervention

Assessments	*N* (T0)	*N* (T1)
Questionnaires	7	7
DIGIKOST-FFQ*	6	5
Treadmill test	7	5
Sit-to-stand test	6	6
Push-up test	6	6
Completed/planned	32/35	29/35
Assessment completion rate	**91%**	**83%**

*N* number; *FFQ* food frequency questionnaire

### Intervention adherence rate

The adherence rate to the patient education sessions was 86% (6 of 7) (Table 3). One participant missed the patient education sessions due to work. Based on information from the exercise logs and HR-sensors, the adherence rates were 69% (58 of 84) for supervised, 54% (42 of 77) for unsupervised and 20% (7 of 35) for voluntary unsupervised exercise sessions (Table 3).

**Table 3:** Adherence to each of the components of the intervention

Intervention component	N/%
Patient education	
Attended/planned sessions	6^1^/7
Adherence rate	86 %
Supervised exercise	
Attended/planned sessions	58^4^/84
Adherence rate	69%
Unsupervised exercise	
Attended/planned sessions	42/77
Adherence rate	54%
Voluntary, unsupervised exercise	
Attended/planned sessions	7/35
Adherence rate	20%
Cognitive behavioral therapy program	
Attended/planned sessions	34^2^/42
Adherence rate	81%
Individual nutritional counselling	
Attended/planned sessions	19^3^/21
Adherence rate	91%

^1^Missed due to work

^2^Number of missed cognitive behavioural therapy program sessions per participant: 1 session (*n* = 1), 2 sessions (*n* = 1) and 5 sessions (*n* = 1)

^3^Number of missed individual nutritional counselling sessions per participant: 2 sessions (*n* = 1)

^4^Number of missed supervised sessions per participant: 2 sessions (*n* = 2), 4 sessions (*n* = 1), 5 sessions (*n* = 2) and 8 sessions (*n* = 1)

Half of the 58 completed supervised exercise sessions were performed with the planned aerobic dose, 15 sessions (26%) with a higher and 14 sessions (24%) with a lower aerobic exercise dose than planned (Supplementary file, Figure S1). Of the 42 completed unsupervised sessions, half were performed with the planned aerobic exercise dose, 13 (31%) with a higher and eight (19%) with a lower aerobic exercise dose than planned (Supplementary file, Figure S1). No adverse events were reported related to the exercise sessions.

The main reasons for non-attendance to the supervised exercise sessions were work and time restrictions, medical advice to refrain from exercise for 2 weeks due to heart problems (*n* = 1) and organizational challenges due to delayed first contact with the local physiotherapist (*n* = 3) (data not shown). Two of seven participants watched the 20-min recorded instruction video including body awareness and attentive presence (data not shown).

The adherence rate of the CBT sessions was 81% (34 of 42 planned sessions) and of the individual nutrition counselling 91% (19 of 21) (Table 3). One participant missed five CBT sessions and found the entire intervention too challenging to combine with full-time work and withdrew from the CBT program and nutrition counselling after one session, but continued the exercise program.

### PREMs

All participants rated the physical exercise as highly useful or useful. Five reported that the CBT program and individual nutritional counselling were highly useful or useful (Table 4). Four participants responded that the total load of the intervention went just fine, while three stated that the load was too much in the beginning. Three of six evaluable participants reported that their fatigue had improved, and four of six reported that they managed their fatigue in a better way at T1 than at T0 (Table 4). Evaluation of the separate parts of the multimodal program are provided in Supplementary file (Table S1 to S4).

**Table 4:** Evaluation of the multimodal program from participants

Question	N
How useful has each of the components been for improving fatigue?	
*Patient education*	
Highly useful	1
Useful	4
Less useful^1^	1
Did not attend	1
*Physical exercise*	
Highly useful	6
Useful	1
Less useful	0
*Cognitive behavioural therapy program*	
Highly useful	2
Useful	3
Less useful^2^	1
Did not attend	1
*Individual nutrition counselling*	
Highly useful	2
Useful	3
Less useful^3^	1
Did not attend	1
How has the total load of the multimodal intervention influenced your fatigue?	
It went just fine	4
It went moderately well	0
It was too much^4^	3
How is your level of fatigue now compared to before you participated in the study?	
Better	3
No change	3
Worse	0
Did not respond to the question	1
How do you manage your fatigue now compared to before you participated in the study?	
Better	4
As before	2
Worse	0
Did not respond to the question	1

^1^Due to technical problems. ^2^Reported to have well-functioning cognitive strategies in the management of fatigue before the intervention. ^3^Reported to have a healthy diet before the intervention. ^4^The first weeks: combination of testing, medical screening, and start-up

### PROMs and physical tests

**Fig. 3:**
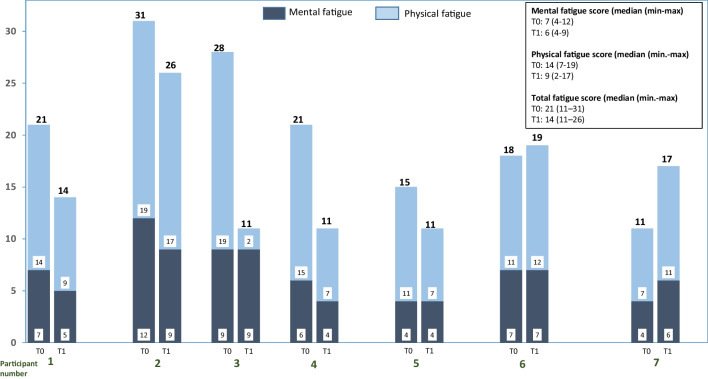
Individual mental and physical fatigue levels at T0 and T1 (*n* = 7)

Five, three and five participants reduced their total, mental and physical fatigue respectively from T0 to T1. Median total fatigue scores were 21 (range 11–31) at T0 and 14 (range 11–26) at T1 (Fig. 3). Median mental fatigue scores were 7 (range 4–12) at T0 and 6 (range 4–9) at T1, while physical fatigue scores were 14 (range 7–19) at T0 and 9 (range 2–17) at T1.

**Fig. 4:**
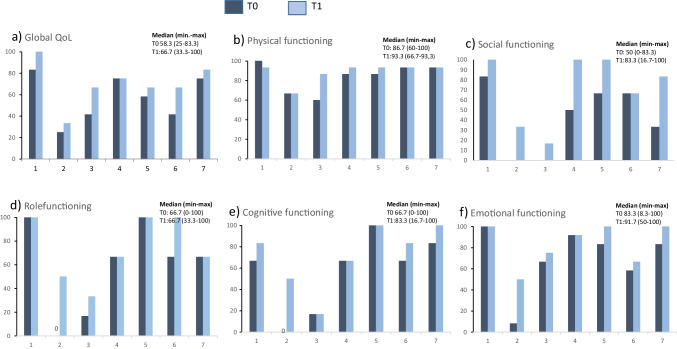
**a–f** individual scores to EORTC QLQ-C30 global quality of life and functioning scales and at T0 and T1 (*n* = 7)

Six participants increased their global QoL, with a median score 58 at T0 (range 25–83) and 67 at T1 (33–100) (Fig. 4a). Individual changes from T0 to T1 for the EORTC functional scales are presented in Fig. 4b–f. The largest improvements were seen for social and cognitive functioning, which increased with more than median 10 points from T0 to T1 (Figs. 4c and 4e).

**Fig. 5:**
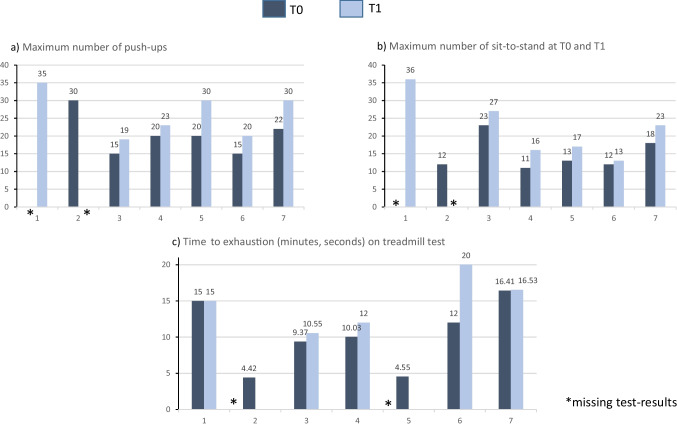
**a–c** Individual physical test results at T0 and T1 (*n* = 7)

All five participants who completed the push-up and sit-to-stand tests at both T0 and T1 improved the number of repetitions (Fig. 5a and b). Three of the five participants who completed the treadmill tests at T0 and T1 increased their duration to exhaustion (Fig. 5c).

**Fig. 6:**
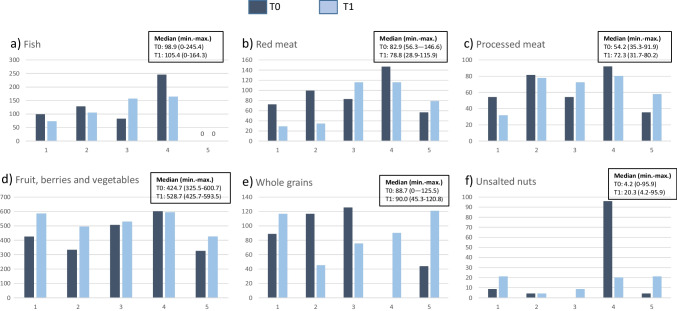
a–f Individual dietary intake (g/d) at T0 and T1 (*n* = 5)

Median intakes of fruit, berries and vegetables, unsalted nuts, whole grains and fish and processed meat increased, while median intake of red meat decreased from T0 to T1 (Fig. 6a–f).

## Discussion

This is the first study to investigate the feasibility of a multidimensional program among lymphoma survivors with CF aiming to reduce their level of fatigue. The main aims with this study were to estimate important parameters needed to design a RCT, such as recruitment and eligibility of participants and adherence rates.

The results show that this multidimensional intervention is feasible and tolerable for this small group of lymphoma survivors with CF. Adherence to the four components varied from 69% (supervised exercise) to 91% (nutritional counselling). The participants rated the program as helpful. The level of total fatigue decreased during the interventional period in five of seven participants.

### Recruitment and retention

Based on the prevalence of CF affecting 25–30% of lymphoma survivors [2, 3, 11], we expected around 12 of the 47 survivors identified by the registry to be eligible for inclusion. With 10 chronic fatigued survivors responding and seven included, we consider the response and inclusion rates as satisfactory, implying that lymphoma survivors with CF are motivated to participate in a multidimensional intervention study like this. Two of the 10 survivors with CF responded after study start. In future RCTs, it will therefore be important to ensure enough response time from invitation letters are sent out to start of the intervention.

There are no directly comparable prior studies to assess response and inclusion rates. However, the inclusion rate in this study (7/47 = 15%) is in accordance with a pilot study from 2003 assessing the feasibility of an exercise programme, using a similar recruitment method [29]. In that study, 63 Hodgkin’s lymphoma survivors were invited by mail to complete a survey including the FQ. A total of 18 survivors reported CF (29%), of whom nine survivors (14%) completed medical and physical tests before entering a 20 weeks aerobic exercise program. A randomized pilot study testing the feasibility and effect of a yoga program versus strengthening exercise in fatigued breast cancer survivors also recruited survivors by mailed invitations, without prior knowledge about their level of fatigue [30]. That study had a response rate of 19% and an inclusion rate of 8%, which is lower than in the present study.

All participants completed the questionnaires at T0 and T1. However, data were missing for the physical tests and DIGIKOST-FFQ either at T0 or T1. For future RCTs, study personnel should have high focus to ensure complete data at all assessment points.

### Adherence to the intervention

Cancer survivors with CF may lack both the capability and motivation to participate in a multidimensional program lasting for longer periods of time. However, participants in the current study showed overall good adherence rates. No one dropped out during the intervention, but one participant withdrew from the nutrition counselling and CBT-based program because all four components and full-time work generated a too high total burden. This might indicate that this intervention is challenging to combine with a full-time job. In a future RCT, it might be relevant to contact eligible working survivors to discuss possible adjustments in work or everyday life that can enable participation, e.g. home office and flexible working hours if possible, and/or support from family or friends on other areas, such as housework and childcare.

Also, given that three of the seven participants found the total load of the intervention too high in the beginning, changes reducing this load should be considered in a future RCT, e.g. ensuring better time for the participants to prepare for the intervention, and possibly spread the different components more out over the 12-week timeline.

Six of seven participants attended the online patient education session, indicating, as shown in previous research [31], that the digital format may have advantages in terms of accessibility, saving the participants from using time and energy travelling.

The participants attended 69% of the supervised exercise sessions, and 75% of these sessions was completed with the planned or a higher aerobic exercise dose than planned. This finding is close to a previously applied feasibility threshold (70%) for an exercise program in patients with metastatic breast cancer, reporting adherence with similar metrics as used here [32]. In two studies of breast cancer survivors with fatigue of unknown duration engaging in individually supervised moderate-high aerobic and/or strength exercise, somewhat higher adherence rates were observed (> 80%) [33, 34]. The lower adherence rate in our study can be explained by that three participants had a delayed start of the exercise program because of late contact with the local physiotherapists. Ensuring that there is enough time from first contact with the physiotherapist to study start is therefore essential in a future RCT.

The adherence to the unsupervised exercise sessions (53%) in our study was higher than reported in previous trials involving home-based exercise for cancer survivors reporting fatigue symptoms (39–44%) [30, 35]. This suggests that the exercise program used in this study was tolerated by the participants, which is in line with their provided feedback. Nevertheless, it is of interest that the number of missed- and dose reduced sessions increased from week 6. As similar observations also has been reported by others, including Scott et al. [36], it will be important in future studies that the study personnel increase their awareness at this time point and keep a close dialogue with the survivors on how to keep the motivation for exercise, e.g. discuss alternative type of activities if indicated or adjust the intensity.

The low number of attended voluntary exercise sessions from week 8 may indicate that two exercise sessions were enough for our seven participants. Based on this observation, we consider to remove this voluntary exercise session in a future RCT. However, as this observation is only based on seven participants, we cannot exclude that a larger sample size would provide a different adherence and a more certain justification for this decision. Furthermore, given that the 20-min pre-recorded instruction were only seen by two participants, exercises for body awareness and attentive presence may be more feasible if provided in a live digital session allowing for interaction between the physiotherapist and participants, such as in the digital CBT group sessions.

The adherence to the CBT-based group program was somewhat lower in our study (81%) compared to two trials that included psychological interventions among cancer survivors with fatigue [34, 37]. Among fatigued colon- and breast cancer survivors, Sandler et al. reported that the survivors received 86% of the total allocated time of individually in-person tailored CBT consultations [37]. Rogers et al. reported a 94% attendance to six in-person discussion groups led by a psychologist, providing behavioural support among fatigued breast cancer survivors participating in an exercise program [34]. One might speculate that the attendance rate would have been higher also in our study if the CBT program was conducted in-person as were done in the two mentioned studies, given that most of the participants would have preferred physical attendance (Supplementary). On the other hand, six of seven participants lived more than one hour from the Cancer Rehabilitation Centre, and it is likely that such a long travel distance would drain energy for the participants. In future studies recruiting fatigued patients from a large geographical area, we consider digital sessions very pertinent.

The high attendance to the individual nutrition counselling and the positive feedback from the participants indicate that nutrition is a relevant and useful part of a multidimensional program targeting CF. The close follow-up from the registered clinical dietitian, who re-scheduled the sessions for participants who for some reason had to cancel, was probably essential to ensure the high attendance rate.

### Changes in fatigue, HRQoL, dietary intake and physical performance and participants’ evaluation of the multidimensional intervention

For five of seven participants the total level of fatigue was reduced, and for six out of seven participants, the global HRQoL score improved. Further, the median total fatigue, social functioning and cognitive functioning scores changed with > 10% of the maximum score from baseline to post-test, which is considered clinically meaningful changes [38, 39]. Similarly, results of physical tests and adherence to the NFBDG seem to improve immediately after the intervention. This indicates that the applied assessments in our study are feasible to detect changes from before to immediately after the intervention. Even in the absence of any reported major improvement, patients seemed to cope better with their fatigue after the intervention, in that the majority responded to handle the fatigue in a better way.

### Strengths and limitations

Strengths of the present study include the multidimensional intervention addressing the complexity of CF and the recruitment of survivors reporting CF using a questionnaire that is validated for measuring CF. The intervention was developed and executed through a close interdisciplinary cooperation, involving both scientists and clinicians. The supervised exercise in the participants’ local environment as well as the digital arrangement enable participants to participate irrespective of their residence. A limitation with the current feasibility study is the recruitment procedure inviting participants by mailed invitations only. We planned to call all the invited survivors after having received the study invitation. In this phone call, we could have identified those without fatigue and those with severe fatigue who were not able to respond or participate in the study. However, the Ethics committee did not approve this. For a feasibility study with only seven participants, we could have invited chronically fatigue survivors from the outpatient clinic at the hospital. In this way, the feasibility of the intervention and assessments could be tested, but not the recruitment procedure. In a future RCT, we plan to include 150 survivors, and in order to reach out to as many survivors as possible in a certain period of time, we found it important to test how many participants we could include in the feasibility study by sending an invitation to all survivors. After conducting this feasibility study, we find the recruitment procedure acceptable, even with the limitations.

Working with the descriptive data from this feasibility study has given us an indication of the amount of data a RCT will generate and that we need to calculate enough time to prepare the data collection and to collect and analyse it. A limitation with the current feasibility study is the lack of registering the exact time used for conducting physical tests and completing the questionnaires. Another limitation is lack of follow-up data; thus, we cannot determine the feasibility of follow-up assessments or whether the observed improvements were maintained for longer than immediately after the intervention. Further, we acknowledge that the time restrictions might have compromised on the adherence to the exercise program.

## Conclusion

A multidimensional intervention including patient education, physical exercise, a CBT-based group program and individual nutritional counselling is feasible among lymphoma survivors with CF. The overall good adherence rate and positive feedback from the participants suggest that all parts of the intervention are relevant and may be helpful to improve fatigue, functioning and QoL. The current feasibility study indicates that it is relevant and feasible to conduct a RCT to assess the effects of a multidimensional intervention on fatigue among lymphoma survivors with CF. However, for an RCT, we consider to make some adjustments, such as ensuring more time for the participants and study personnel to prepare for the intervention, reducing the number of exercise sessions and excluding or changing the delivery format of the pre-recorded exercises for body awareness and attentive presence.

### Supplementary information

ESM 1 (DOCX 22 kb)

## Data Availability

Derived data supporting the findings of this study are available from the corresponding author [SKHB] on request.
