# Supplementary material for: The feasibility of a multidimensional intervention in lymphoma survivors with chronic fatigue
Source: Support Care Cancer. 2023 Dec 14;32(1):22. doi: 10.1007/s00520-023-08204-5 (PMC10721709; doi:10.1007/s00520-023-08204-5)
Supplement: Supplementary file 1 — (DOCX 22 kb) [file 520_2023_8204_MOESM1_ESM.docx]

### Supplementary file

**Table S1)** Evaluation of the patient education

| **Lymphoma survivors (n=7)** | |
| --- | --- |
| **How did you experience to attend the digital patient education?** | |
| It went well | 6 |
| It did not go well | 0 |
| Did not attend | 1 |

**Table S2)** Evaluation of the exercise program

| **Lymphoma survivors (n=7)** | |
| --- | --- |
| **How did you experience the intensity on aerobic exercise program?** | |
| *Week 1-2 (adaption period)* | |
| Too easy | 4 |
| Adequate | 3 |
| Too heavy | 1 |
| *Week 3-6 (70-75% HFpeak)* | |
| Too easy | 3 |
| Adequate | 3 |
| Too heavy | 1 |
| *Week 7-12 (80-85 % HFpeak)* | |
| Too easy | 0 |
| Adequate | 6 |
| Too heavy | 0 |
| Missing | 1 |
| **From week 7-12, you were instructed to have a heart rate of 80-85 % of HRmax. How did this correspond to your subjective experience?** | |
| I exercised with lower intensity | 1 |
| This corresponds well with my experience | 5 |
| I exercised with higher intensity | 1 |
| **Were the exercises in the strength training program ok to perform?** | |
| No | 2* |
| Yes | 5 |
| **How did you experience the intensity of the strength training program?** | |
| Too easy | 0 |
| Adequate | 7 |
| Too heavy | 0 |
| **How did you experience to increase the total number of sets in the strength training program from week 4?** | |
| It went just fine | 1 |
| It went moderately well | 6 |
| It did not go well | 0 |
| **How did you experience the total duration of the exercise session?** | |
| Too short | 1 |
| Appropriate | 5 |
| Too long | 1 |

*Problems with squats due to knee-pain (n=1), cannot do upper body exercises due to frozen shoulder (n=1).

**Table S3)** Evaluation of the cognitive behavioral therapy program

| **Lymphoma survivors (n=7)** | |
| --- | --- |
| **Would you prefer physical or digital attendance to the CBT group program?** | |
| Physical | 4 |
| Digital | 2 |
| Don’t know | 0 |
| Did not attend | 1 |
| **How satisfied were you with the CBT-program overall?** | |
| Very satisfied | 4 |
| Satisfied | 2 |
| Less satisfied | 0 |
| Did not attend | 1 |

CBT: cognitive behavioural therapy program

**Table S4)** Evaluation of the nutrition counselling

| Lymphoma survivors (n=7) | |
| --- | --- |
| **Have you implemented any changes in your diet as a result of the counseling?** | |
| Yes* | 4 |
| No | 2 |
| Did not attend | 1 |
| **Could the nutritional counseling been in groups instead of individual?** | |
| No, I prefer individual | 6 |
| Group sessions would have been fine for me | 0 |
| Did not attend | 1 |

*More fruit/vegetables, fish, wholegrain, more dairy products, higher meal frequency, started to eat breakfast

**Figure S1**: adherence to the 23 planned exercise sessions. Session 1, 3, 5, 7, 9, 11 , 13, 15, 17, 19 , 21 and 23 were supervised, session 2, 4, 6,8, 10, 12, 14, 16, 18, 20 and 22 were unsupervised.

Green bars: number of participants that completed the session with the planned aerobic exercise intensity and volume as planned; Yellow: number of participants that completed the sessions with a lower aerobic exercise intensity and volume than planned; Blue: number of participants that completed the session with a higher aerobic exercise intensity and volume as planned; Red bars: number of participants that missed the session;
